# Proton Pump Inhibitor Use in Older Adult Patients with Multiple Chronic Conditions: Clinical Risks and Best Practices

**DOI:** 10.3390/jcm14155318

**Published:** 2025-07-28

**Authors:** Laura Maria Condur, Sergiu Ioachim Chirila, Luana Alexandrescu, Mihaela Adela Iancu, Andrea Elena Neculau, Filip Vasile Berariu, Lavinia Toma, Alina Doina Nicoara

**Affiliations:** 1 Faculty of Medicine, Ovidius University of Constanta, 900470 Constanta, Romania; lauracondur@yahoo.com (L.M.C.); sergiu.chirila@univ-ovidius.ro (S.I.C.); alinadnicoara@yahoo.com (A.D.N.); 2 ”Sf. Apostol Andrei” Emergency County Hospital, 900591 Constanta, Romania; tomalaviniamed@yahoo.com; 3 Department of Internal Medicine (Cardiology, Gastroenterology, Hepatology, Rheumatology, Geriatrics), Family Medicine, Labor Medicine, “Carol Davila” University of Medicine and Pharmacy, 020021 Bucharest, Romania; 4 Department of Fundamental, Clinical and Prophylactic Sciences, Transylvania University of Brasov, 500019 Brasov, Romania; andrea.neculau@gmail.com; 5 Department of Plastic Surgery, Regional Institute of Oncology, 700361 Iasi, Romania; filip.berariu@gmail.com

**Keywords:** aging, chronic diseases, comorbidities, proton pump inhibitors, side effects, benefits, risks

## Abstract

**Background and objectives:** Life expectancies have increased globally, including in Romania, leading to an aging population and thus increasing the burden of chronic diseases. Over 80% of individuals over 65 have more than three chronic conditions, with many exceeding ten and often requiring multiple medications and supplements. This widespread polypharmacy raises concerns about drug interactions, side effects, and inappropriate prescribing. This review examines the impact of polypharmacy in older adult patients, focusing on the physiological changes affecting drug metabolism and the potential risks associated with excessive medication use. Special attention is given to proton pump inhibitors (PPIs), a commonly prescribed drug class with significant benefits but also risks when misused. The aging process alters drug absorption and metabolism, necessitating careful prescription evaluation. **Methods:** We conducted literature research on polypharmacy and PPIs usage in the older adult population and the risk associated with this practice, synthesizing 217 articles within this narrative review. **Results:** The overuse of medications, including PPIs, may lead to adverse effects and increased health risks. Clinical tools such as the Beers criteria, the STOPP/START Criteria, and the FORTA list offer structured guidance for optimizing pharmacological treatments while minimizing harm. Despite PPIs’ well-documented safety and efficacy, inappropriate long-term use has raised concerns in the medical community. Efforts are being made internationally to regulate their consumption and reduce the associated risks. **Conclusions:** Physicians across all specialties must assess the risk–benefit balance when prescribing medications to older adult patients. A personalized treatment approach, supported by evidence-based prescribing tools, is essential to ensure safe and effective pharmacotherapy. Addressing inappropriate PPI use is a priority to prevent potential health complications.

## 1. Introduction

Older adult patients, defined as adults over 65 years of age (UN and WHO) [1,2], require special medical attention and represent a challenge in any specialty. In fact, older adult patients have, most of the time, multiple comorbidities (such as cardiovascular diseases, metabolism diseases, osteo-articular degenerative diseases, and digestive diseases), leading to a higher degree of frailty and thus needing an integrated, interdisciplinary approach from a complex medical team. Aging is a complex, multifactorial phenomenon that yet remains incompletely understood. It involves a physiological, progressive, and widespread alteration in all body functions. As we age, we witness a process of decreasing adaptation to stress, accompanied by an increase in the incidence and prevalence of chronic diseases. Although, to a certain extent, aging is a physiological, irreversible process, it is also highly individualized, and the pace at which it occurs is very important [1,2]. The presence of several comorbidities, accompanied by polypharmacy, is another particularity of these patients. Polypharmacy refers to the use of multiple medications, often defined as five or more medications per patient. Existing data suggest that approximately 30 to 50% of older adults are affected by it [3,4], increasing the risks of adverse drug events. At this stage in life, there is a tendency for the uncontrolled administration of certain therapies (medication for existing comorbidities, over-the-counter medications such as nonsteroidal anti-inflammatory drugs, or dietary supplements), on the patient’s own initiative (self-medication), prescribed by physicians, or even a pharmacist’s recommendation. One reason is the idea of taking preventive measures for the possible side effects of chronic medication. While, in specific cases, these precautions are necessary, most of the time, these represent over-medication, which can lead to unwanted effects that are often ignored. Drug interactions, including those with food supplements, can reduce or delay the effectiveness of essential medications.

The effects of the physiological aging process are sometimes also reflected in the need to adapt the doses of drugs to the decrease in hepatic or renal function, metabolization, or the elimination of drugs. Therefore, the clinical and therapeutic approach to chronic patients with multiple comorbidities is an extremely complex and profound medical act. One category of such drugs, which is associated with clear recommendations and certain benefits when the rules of prescription are followed but which has been shown, in practice, to often be exposed to overuse, is proton pump inhibitors (PPIs).

## 2. Aging of the Gastrointestinal System

**Figure 1:**
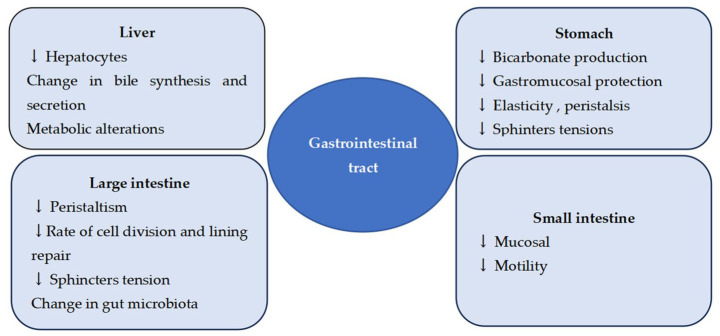
Physiological change in older patients [1,2,3,4]. (↓—decreases).

As it is already known, the gastrointestinal system plays an essential role in the absorption and metabolism of drugs. Thus, in the case of chronic medication for different pathologies, the risk of side effects may increase at this level. On the other hand, the gastrointestinal tract physiologically undergoes a series of processes that influence the overall health of each individual. Physiologically, the aging process affects all the functions of the gastrointestinal system (reduced gastric acid secretion), gastrointestinal motility (delayed gastrointestinal motility), the digestive secretion of enzymes and hormones (decreased mucosal repair mechanisms), and digestion and absorption processes [1,5]. Thus, some parameters increase, others decrease, or others do not even show changes with age [1] as detailed in Figure 1. These aspects should be known, however, by all clinicians and taken into account when talking about the clinical and therapeutic approach to an older adult patient. The aging of the gastrointestinal process appears not to be related to a decrease in gastric acid secretion, with studies demonstrating that this process is related to the presence of Helicobacter pylori [6,7]. A decrease in gastric peristalsis, a decrease in intestinal motility, which favors the increased elimination of gastric contents, and the appearance of constipation (a symptom frequently encountered among older adults). Physiological changes in immunity lead to a decrease in the immune response in the intestinal mucosa, which is associated with an increased risk of infection among older adults [1]. A decrease in plasma ghrelin (the “hunger” hormone) could explain the concept of the “anorexia of ageing” [8], and a decrease in Cyp450 (a cytochrome essential for the metabolization of many drugs) could exert an important influence [1]. On the other hand, we are witnessing an increase in some parameters: gastroesophageal reflux, gastric evacuation time, which increases the susceptibility to gastric and esophageal pathologies, and an increase in the susceptibility to gastrointestinal neoplasia among older adults (due to “increased cell proliferation secondary to intestinal lesions”) [8]. The digestive properties of the intestine, transit time, and gastrin secretion do not change [1,8]. Aging induces multifaceted alterations in gastrointestinal function, including diminished digestive enzyme production, may favor the development of specific pathologies. These may be accentuated in the context of other comorbidities, including the development of protein–caloric malabsorption (PCM) [1,5,8]. This (PCM) is considered a pathologic process facilitated via the intestinal bacterial overpopulation, which is secondary to gastric hypochlorhydria or jejunal diverticulosis and the atheromatosis of the superior mesenteric artery [8]. Gastric atrophy is common among older adults (prevalence of 50–70% in people over 80 years of age) and is nowadays considered “a pathologic gastric aging process” [8], in which Helicobacter pylori infection may play an important role. Studies have shown that “the eradication of this bacteria in the older adults can reverse the process of gastric atrophy, regardless of its degree” [8]. This may explain the increased susceptibility of older adults to certain pathologies, such as gastric ulcer, atrophic gastritis, peptic ulcer, or the development of gastrointestinal side effects in certain medications. Therefore, the management of gastrointestinal diseases among older adults presents significant differences compared to younger age groups, involving significant challenges for the current clinical practice that are especially significant in the context of increased PPI consumption among older adults. Moreover, the consumption of PPIs has increased rapidly in recent years, not necessarily in the context of gastrointestinal disease but in the light of comorbidities and polypharmacy, which are realities for this age group [9]. Studies show an inappropriate use of PPIs in 40–65% of patients, mainly due to prescriptions for a long time and without a clear indication [10]. Considering these aspects, the possibility of adverse effects, ineffective administration, and unnecessary additional costs [11] represents a serious concern.

It is important to acknowledge that significant initiatives are present throughout the world for “stomach health”, such as the efforts of different organizations, which came together under the Healthy Stomach Initiative [12]. Its main purpose is to promote and disseminate knowledge, not only about the healthy stomach but also about related conditions, with the purpose of preventing and correctly treating gastric pathology. To achieve this, efforts for developing scientific research in the field are undertaken through this initiative. At the same time, for a wider reach in the general population, World Stomach Day was set for the 2nd of October.

## 3. Material and Methods

A narrative review was selected as the preferred methodology because of the need for a broad and integrative synthesis of the existing knowledge on this complex topic. This narrative review synthesizes current evidence on PPI use in older adults with multiple chronic conditions. A detailed scoping or systematic review was beyond the purview of this article, but we have included a structured discussion of key findings and recommendations based on high-quality studies. Also, given the heterogeneity of study designs, populations, and outcomes within the literature, a narrative approach allowed for a comprehensive and flexible discussion that captured a wide range of relevant clinical perspectives. The review focused on older adult patients (aged > 65 years) with comorbidities, specifically examining the use of proton pump inhibitors (PPIs). Emphasis was placed on patterns of use, associated with adverse reactions, and medical interactions. The primary objective of this study was to conduct a preliminary evaluation of the risk–benefit profile associated with PPI use among older adult patients in primary care settings.

By identifying usage patterns, related clinical outcomes, and potential risk factors, this review aimed to contribute to the development of safer, more judicious prescribing practices and to support the future formulation of clinical guidelines tailored to this vulnerable population. To review the existing literature, a systematic search was conducted manually by two researchers using the PubMed and Web of Science databases. The search strategy included the following keywords: “proton pump inhibitors” or “PPIs” or “omeprazole” or “pantoprazole” or “esomeprazole” or “lansoprazole” or “rabeprazole” and “older adults” or “aged” or “geriatric patients” and “comorbidities” or “chronic disease” and “therapeutic use” or “efficacy” or “safety” or “interactions” or “long-term use”. Only studies published after 2014, in English, were considered. Studies focusing exclusively on the general adult population were excluded. In total, 217 articles were screened and synthesized. Full texts were assessed for eligibility following initial screening.

To assess the clinical risks and benefits of PPIs, we included studies across various research designs (e.g., randomized controlled trials, observational studies, and meta-analyses). However, special emphasis was placed on high-quality meta-analyses and systematic reviews, as they provide a higher level of evidence compared to individual case reports or small-scale observational studies.

During the analysis, recurring themes began to emerge, suggesting that data saturation had been reached prior to analyzing all eligible articles. Study quality was assessed based on research design. Meta-analyses and systematic reviews were prioritized due to their robust methodology, while case reports and smaller observational studies were used to highlight specific risks or unique clinical scenarios. The findings were synthesized in a descriptive format, highlighting current knowledge, clinical implications, and recommendations for primary care providers.

## 4. Proton Pump Inhibitors (PPIs)

PPIs are a widely recommended class of drugs, practically the most widely prescribed in gastroenterology, though not the only therapeutic option. According to the FDA Classification (2015), we know the first-generation PPIs, Omeprazole (it is the best studied), Pantaprazole, Lansoprazole, and Rabeprazole (it still involves many unknowns, but there are studies that consider it appropriate for older adult population, without the need to adjust the dosage) [13], and the second-generation PPIs: Esomepromazol, Dexlansoprazole, and Tenatoprazole. Also, Ilaprazole, a new generation PPI, shows promising results in the treatment of gastroesophageal reflux disease, peptic ulcer, and duodenal ulcer [14].

A systematic review of trends and practices of PPI prescriptions patterns and consumption trends in the general population was published in 2023 [15]. In this analysis, studies conducted between 1988 and 31 March 2023, were searched for PPIs consumption among the population above 18 years of age. The study identified 65 articles, with 28 million PPIs users in 23 countries, of which 37% were over 65 years old [15]. At the same time, studies have shown that some PPIs (Omeprazole) are among the top-prescribed medicines, occupying second place in the UK and eighth place in the USA [15]. The question that arises in this context is when to start prescribing and administering PPIs and when to stop.

Biochemically, PPIs are derivatives of the heterocyclic organic molecule benzimidazole that inhibits proton pumps. Proton pumps, also known as hydrogen–potassium–adenosine triphosphatase (H,K-ATPase), are cell-membrane transport structures that produce HCL in the stomach [15,16] through a process of ion exchange [17]. The proton pump is a heterodimeric protein, being produced via two genes, namely ATP4A, which encodes the α subunit, and ATP4B, which encodes the β subunit [18].

Another important pharmacological aspect is that PPIs are metabolized hepatically via the cytochrome CYP2C19. Given the genetic polymorphism involving 21 mutant alleles, the effects of CYP2C19 on PPIs metabolization rate can differ [19]. This pharmacogenetic aspect is crucial to study, as it may be associated with PPI exposure, efficacy, and adverse effects. Essentially, patients can be classified into two groups based on CYP2C19 phenotypes: extensive metabolizers and poor metabolizers of PPIs [20]. This classification helps explain the diverse individual variations in PPI pharmacokinetics and pharmacodynamics within the general population. Research indicates a significant association between the genetic polymorphism of CYP2C19 and the therapeutic response to PPIs [20].

Studies show that PPIs irreversibly inactivate the proton pump in gastric parietal cells, reducing daily HCL production by 80–95%. PPIs have long-lasting antisecretory effect, inhibiting gastric acid secretion, regardless of the stimulus. By lowering the acidity level of the gastric juice, they play a crucial role in treating ulcerative disease. Importantly, their action persists for at least 24 h, thus offering a significant therapeutic advantage. Reports also note that an inhibition level of approximately 20–26% is still present even one week after the discontinuation of the treatment, making the administration in a single daily dose optimum, with minimum risks for adverse effects [16]. Studies also indicate that PPIs are effective for the treatment of ulcerative ulcer disease, with significant improvements or even complete healing. The mechanism of action involves not only reducing gastric acidity but also enhancing microcirculation at the site of the ulcer lesions, further promoting recovery [16]. Considering these aspects, clinical guidelines establish indications for the prescription and use of PPIs in the short term (4–8 weeks) and long term (>8 weeks) [21]. Thus, one of the recommendations for long-term use is “chronic use of NSAIDs in people at moderate or high risk of bleeding (age over 65 years, high doses of NSAIDs, history of ulcer, concomitant corticosteroid medication, antiplatelet or anticoagulant” [21]. Considering the current knowledge, the benefits of PPIs are certain and indisputable, and they are considered to be safe and highly effective [22]. The condition is that PPIs are administered correctly, in terms of indication and time, according to guidelines and expert recommendations [23]. Among older adults, we are witnessing overprescription or overuse, which should be a warning sign [24]. Studies show that the use of PPIs in older adults for a period longer than 8 weeks, outside the existing recommendations, is not recommended [25]. Unfortunately, practice shows us otherwise, with patients using PPIs for months and even years without a clear recommendation, which can certainly lead to the development of effects that might overcome the benefits of the treatment [26].

## 5. Adverse Effects of Proton Pump Inhibitors

Published data suggest the potential long-term adverse effects (defined as years of continuous use) of PPI therapy among older adults [27,28]. Following research in the literature, we offer an update of the studies from recent years that we consider necessary to be taken into account when prescribing or using PPIs in the medium or long term.

### 5.1. Hypomagnesemia

Hypomagnesemia is a relatively rare adverse reaction, but it is not to be neglected. The hypothesized mechanism for decreased magnesium levels is related to the decrease in the active intestinal absorption of magnesium via transient protein channels (TRPM6/7), due to lower extracellular proton stimulation following PPI treatment [16,29]. Hypomagnesemia, when severe, can lead to serious complications such as tetanus, convulsions, delirium, and cardiac arrhythmias [30]. The recommendation is to perform mandatory serum magnesium testing in patients with risk factors such as malnutrition, renal failure, etc. At the same time, it is useful to monitor magnesium in patients who receive PPI treatment for a long time and at a high dosage [31].

### 5.2. Deficiency in Vitamin B12, Vitamin D, Vitamin C, Iron, or Calcium

Excessive PPI consumption has been associated, over time, with an increased risk of vitamin and mineral deficiencies, especially in older adult patients, malnourished, and/or hemodialysis patients. The existing studies (most of them of lower quality—case reports and cross-sectional observational studies) are related to the relationship between vitamin B12 (cobalamin) and PPIs [21]. This conclusion is that the excessive use of PPI, via the exaggerated decrease in gastric acidity, could lead to a decrease in vitamin B12 absorption and bacterial overpopulation in the digestive tract. This risk increases with age and could even explain the occurrence of atrophic gastritis and achlorhydria. Of course, in order to implement a guideline recommendation regarding the clinical approach to this subject, more studies are needed [32].

### 5.3. Osteoporosis and Risk of Fracture

The mechanism is a reduction in calcium absorption through decreased gastric acidity, on the one hand, and secondary hyperparathyroidism and the alteration of enzymes involved in bone remodeling (H-K-ATP-osteoclastic acidase) on the other hand [33]. It is important to note that there are a number of associated factors that increase the risk of osteoporosis, such as long-term treatment with PPIs (more than 1 year), high doses of PPIs (more than 30 mg/day), associated therapies (corticotherapy, anti-Parkinsonian, and antidepressants), or various comorbidities. The recommendation is to continue treatment with PPIs if the therapeutic indication is correct and necessary [15,34].

### 5.4. Increased Risk of Cardiovascular Complications and Death

This can be observed not only among older adults but also in the general population. There are studies confirming this risk, which seems to be higher for patients with DZ type 2. One such study was published in 2023 in the Clinical Journal of Endocrinology and Metabolism [35], and another was published in 2024 [36] in Drugs & Aging. Other studies indicate a significantly increased risk of cardiovascular disease and heart failure when PPIs are used for more than five years (cumulative exposure) [37]. The 2024 study [36] introduces an interesting element in that “a higher prevalence of comorbidities and medications” was found in PPI-using patients compared to the non-PPI-using group. This confirms that excess PPI use is especially present among patients with Crohn’s disease and comorbidities. The findings also confirmed the “increased risk of cardiovascular events and all-cause mortality in a large population of adults with DZ exposed to PPIs” [36,37]. Another interesting study [38], published in 2024, highlights a possible association between PPI consumption and the risk of QT prolongation, including the risk of even torsade pointes. This was observed mainly in critically ill patients. It seems that Lansoprazole and Pantaprazole present a higher risk compared to Omeprazole. Therefore, especially for critically ill patients, the combination of PPIs and other QT prolonging drugs should be avoided [38,39].

### 5.5. Impairment of Renal Function

**Figure 2:**
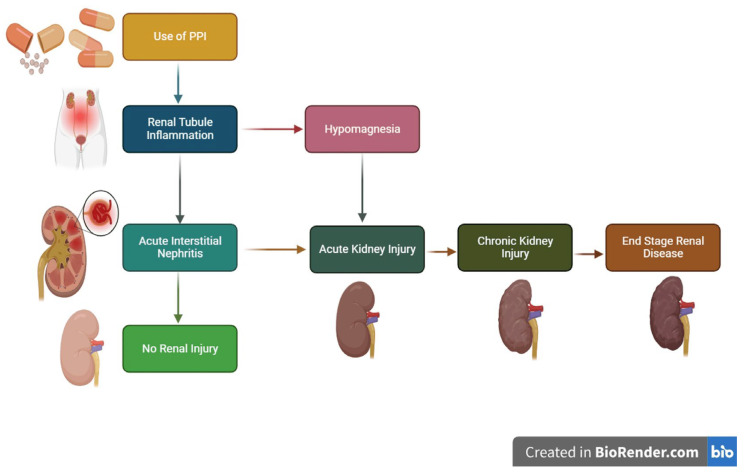
Pathophysiology of PPI and CKD progression [reproduced from Parmar et al., Cureus Journal of Medical Science, published by Cureus] [41].

There is currently research drawing attention to potential risks in this direction, namely the development and/or progression of chronic kidney disease (CKD) following the excessive and uncontrolled use of PPIs [40] as illustrated in Figure 2. A meta-analysis published in December 2023, which included a review of articles published in PubMed, Pub Med Central (PMC), and Google Scholar over a 10-year period (2013–2023) on this topic, highlighted the existence of a possible causal relationship between PPIs, respectively the presence of acute kidney injury such as a decreased glomerular filtration rate (GFR) or the development of CKD. Potential mechanisms through which PPIs could cause nephrotoxicity are also highlighted [41].

At the same time, it appears that PPIs are among the most common causes of drug induced acute interstitial nephritis (AIN) globally [41]. Collected data suggest that the risk is not dose-dependent, and the onset of AIN can be anywhere between 10 weeks and up to 9 months after the start of treatment. Also, the risk of developing CKD is mentioned, but for older adult patients with chronic pathologies, it is mainly aimed at worsening the evolution of CKD, as described in a review of observational studies [42]. The conclusion is that studies are still needed to carefully evaluate the benefit–risk ratio of PPIs, especially for patients with pre-existing kidney disease, a situation often encountered among older adult patients with comorbidities.

### 5.6. The Infectious Risk of Overuse of PPIs

This can be explained, on the one hand, by the lowering of the protective gastric acid barrier caused by the overconsumption of PPIs and, on the other hand, by the decrease in gastric acidity, which leads to an increased concentration of bacteria and an increased risk of bacterial aspiration [43]. In this context, studies show a moderate risk of pneumonia, about 1.89 times higher, that is more evident for those receiving high doses in the short term [44]. Also, an increased risk of spontaneous bacterial peritonitis for patients with liver cirrhosis (even when used as recommended) was observed [45], along with an increased risk for bacterial intestinal overpopulation syndrome [BIBO], enteric infections, and acute infectious diarrhea due to the overuse of PPIs [45]. One recommendation for preventing the risk of acute diarrhea is to stop PPIs when traveling to an area endemic for enteric infections. At the same time, Clostridium difficile infection may also have an increased incidence in the context of prolonged PPI use, according to studies that confirm this association [45].

As a mechanism, the association of antibiotic consumption with PPIs can further reduce the gastric acidity, reaching hypo-acidosis, which in turn favor the survival of spores and toxins of Clostridium difficile. In the context of the decreased defensive function of the organism, there is a high risk for the colonization of the digestive tract with pathogenic germs [33]. The existing data suggest that the severity and complications of pseudomembranous colitis are not PPIs that are dose-dependent or duration-dependent, especially in older adult people. Nonetheless, systematic reviews indicate that PPIs usage is considered a risk factor for the recurrence of Clostridium difficile infection [45].

### 5.7. PPI and the Risk of SARS-COV-2 Infection [46]

This is also a topic of discussion in the wake of the recently ended pandemic. Studies have started since the SARS epidemic in 2003 and have followed the influence of gastric pH on SARS-COV-1 infection. Subsequently, studies have focused on the role that PPI consumption may have on the severity of SARS-COV-2 infection. As a mechanism, the authors hypothesized that SARS-COV-2 can enter the body not only at the respiratory level but also “via Angiotensin converting enzyme 2 receptors in the intestinal tract, invading enterocytes and replicating at this level” [46]. Evidence in this sense would be the detection in the feces of viral DNA or even live virus [46]. As a consequence of this mechanism, in addition to the presence of local symptoms (gastritis, colitis, and enteritis), there is also the risk of spreading the infection, favoring inflammation in other organs and systems, including respiratory, through the intestine–plantar axis [46]. However, the studies have shown contradictory associations, but a meta-analysis conducted in 2022 attempted to make a “holistic assessment of the effect of PPI use on the incidence and prognosis of COVID-19 and to draw an evidence-based conclusion” [47]. The conclusion was that the use of PPIs results in a “nominally increased but statistically significant risk of developing COVID-19, but also an increased risk of severity and mortality in patients with SARS-COV 2 infection” [48]. Of course, these risks increase for older adult patients with multiple comorbidities. The issue is still a topical one and certainly requires further research.

### 5.8. The Role of PPIs on the Intestinal Microbiota

Under the influence of inadequate and prolonged administration, data suggests that PPIs can affect the oral and intestinal microbiota, thus increasing the risk of developing pathologies or exacerbating other existing diseases, such as functional dyspepsia, SIBO, or Clostridium difficile infection [49,50,51]. There are studies that show differences between those who receive and those who do not use PPIs in that “a less healthy microbiome” has been found with PPI treatments [52,53,54]. As a mechanism, on the one hand, there is talk of “an indirect impact of PPIs mediated by suppression of gastric acid” and, on the other hand, of “the direct impact of PPIs on the gut microbial composition by inhibiting specific commensal gut bacteria” [49,55,56]. Intensive research is needed to clarify these clinical findings, i.e., the influence that PPIs may have on the gut flora [57].

### 5.9. The Risk of Cancer

The potential cancer risk associated with PPI long-term use has increasingly become a topic of concern in discussions about their safety [58,59]. High-quality studies in the literature analyze the possible causal relationship between the long-term administration of PPIs and the risk of developing neoplastic disease, namely gastric, esophageal, colorectal, or pancreatic cancer [60,61]. The mechanism is still uncertain, but there is talk about the role that “alteration of gastric pH and microbiome, vitamin and mineral malabsorption, hypergastrinemia, pro-liferation of enterochromatophin-like cells”, etc., could play in this context [62]. For example, there are studies showing that PPIs decrease gastric acid production, which accelerates gastrin secretion, a mechanism that explains the increased risk of gastric cancer [62]. A meta-analysis of epidemiological studies from 2022 found an 80% higher risk of gastric cancer for PPI users compared to non-users, with higher risk associated with more than three years of PPIs administration and increased age [63]. There are also studies showing that “nitrosamines and changes in the gut microbiome may influence the increased risk” [63]. There is less evidence for colorectal cancer risk (controlled studies).

### 5.10. The Risk of Dementia and Neurocognitive Disorders

**Figure 3:**
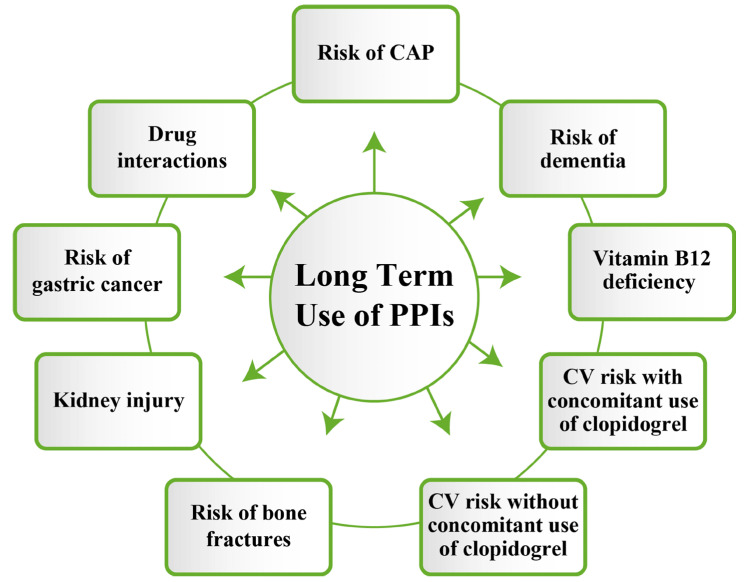
Potential safety concerns associated with long-term use of PPIs [reproduced from Bhathagar et al., Cureus Journal of Medical Science, published by Cureus] [70].

This represents an attractive topic of research in the later years. Here again, studies are still controversial, but there is already an indication that “PPIs may increase the risk of dementia” [64]. On the one hand, there is evidence to support this, but the question arises as to whether “the timing of exposure or age at diagnosis of dementia may influence the risk” [65]. One such study was conducted by Danish researchers on a cohort of 1,983,785 people aged 60–75 years, over a period of 18 years (2000–2018). The results of a nationwide study confirmed the relationship between PPI consumption and dementia, regardless of the timing of the initiation, for people younger than 90 years old [64]. At the same time, it was also shown that this causal relationship cannot be sustained after the age of 90 years [66]. The precise mechanism underlying this causality is still unknown. On the one hand, there is talk that PPIs may alter the pH in brain cells, which could favor the accumulation of beta-amyloid peptide, which plays an essential role in the development of Alzheimer’s dementia [67]. On the other hand, the relationship between the gut–brain axis, PPIs, and microbiota is also being discussed [67,68]. There are studies that support this theory, highlighting the impact that the excessive use of PPIs can have not only on the brain–gut–microbiota axis but also in the production of intestinal dysbiosis, which could favor the development of neurodegenerative diseases and cognitive disorders, including dementia [67,68]. To these theories is added the micronutrient deficiency (vitamin B12, vitamin D, magnesium, iron, and calcium), which has been shown to accompany the excessive consumption of PPIs, favoring the development of cognitive disorders. While further studies are certainly needed, existing evidence suggests a potential susceptibility to a causal relationship between excessive PPI use and dementia.The recommendations are to be careful and cautious in prescribing PPIs, especially to geriatric patients and/or for longer periods than the existing indications [67,69]. Many of the long-term consequences associated with PPI use, as reported in the aforementioned studies, are illustrated in Figure 3.

## 6. Proton Pump Inhibitors and Drug Interactions

Although PPIs are one of the most prescribed drugs worldwide (over 113 million prescriptions), unfortunately, there is no discussion in clinical practice about possible “drug interactions” that may exist and that may affect patients in general and older adult patients, in particular older adult patients with comorbidities [71]. Through their action, PPIs modify the absorption of some drugs, for example, tyrosine kinase inhibitors (Danetimib and Erlotinub), protease inhibitors (Atazanavir and Indinovir), and antifungals, commonly used in practice, such as Ketoconazole or Itroconazole (Table 1). Also, the use of PPIs in combination with oral iron preparations is considered one of the most common interactions (PPIs modify iron absorption). In this context, because PPIs are metabolized in the liver via CYP2C19 and CYP3A4 isoenzymes, this could influence the action of other drugs that are metabolized via the same enzymes. Studies show that, of all PPIs, Omeprazole, Esomepromazole, and Lansoprazole exert greater inhibitory action on CYP2C19 compared to Pantoprazole. Also, Omeprazole plays a greater role in inhibiting CYP3A4 than other PPIs. Rabeprazole, through its thioether metabolite competitively inhibits both CYP2C19 and CYP3A4 and may still perpetuate these interactions [71]. The consequence of this mechanism is an increase in the concentration of the drug in question. In this category are included Phenytoin, Diazepam (CYP2C19), Carbamazepine, and Cyclosporine (CYP3A4). An important interaction for the frequency with which it is encountered in medical practice is that between PPIs (especially Omeprazole) and Clopidogrel. PPIs, by inhibiting CYP2C19, may reduce the metabolization of Clopidogrel precursors, reducing its clinical efficacy [72]. When Prasugrel (used for the treatment of pulmonary hypertension) or Ticagrelor (an antithrombotic, mainly used in the prevention of atherothrombotic events for patients with acute coronary syndrome) are used together with PPIs, these do not interact [73]. Also, PPIs delay the elimination of methotrexate, a commonly used combination [71]. The recommendation of specialists is, when it is necessary to administer these drugs together with PPIs, to ensure a distance of several hours between them and PPIs, as well as the use of Pantoprazole, which is less prone to drug interactions [71,74].

A new class of medications that could be recommended in situations where proton pump inhibitors (PPIs) are less effective, particularly due to CYP2C19 metabolism, are potassium-competitive acid blockers (P-CABs). Although some representatives of this class, such as Vonoprazan, have been approved in the United States as part of combination therapy for the eradication of Helicobacter pylori infection, its efficiency in clinical studies, for similar indications with PPIs, requires further investigations, especially for potential long-time adverse reactions [75].

## 7. Proton Pump Inhibitors Treatment Approach for Older Adults Patients with Comorbidities

The geriatric patient is, as we have already shown, the most susceptible to adverse reactions and to the development of new pathologies if the administration of medication for different chronic diseases is not approached in an integrative and holistic manner. The recommendation of geriatric specialists is to conduct a medication review for each patient in order to avoid polypharmacy, an excess of drugs and supplements, as well as to avoid as much as possible and/or ensure the early detection of adverse effects. In this context, the International Geriatrics Societies recommend a series of tools to enable this “periodic medication inventory”, which should be an essential component of geriatric assessment. We are thus talking about a series of criteria that are quantified in different rating scales, criteria that “are prescribing quality indicators that each clinician can use in prescribing a prescription, which are not drug or disease specific, but are based on the clinician’s medical expertise and focus on the patient” [76]. The main tools used in practice are as follows.

A. The Beers criteria—the most widely used at the moment—are recommended by the American Geriatrics Society, were first introduced in 1991, were revised subsequently (last revision in 2023), and represent a “list of recommendations that help health care providers safely prescribe medications for adults over 65 years of age” [76]. They are revised every 3 years and are a very useful tool for medical practice. Regarding PPIs, the Beers 2019 criteria, revised in 2023, recommend “avoid use for more than 8 weeks, except in high-risk patients, namely those receiving chronic NSAIDs, oral corticosteroids, erosive esophagitis, Barrett’s esophagus, hypersecretory conditions or those with other proven indications for PPIs”. It also recommends that “PPI prescriptions should be reassessed at least annually for appropriateness” and “PPIs should be deprescribed when no longer needed” [8,11]. These recommendations are also supported by the American Gastroenterological Association. The Beers criteria, revised in 2023, also bring to attention the possible adverse effects of PPIs, namely the risk of Clostridium difficile infection, osteoporosis, and fractures, with high levels of evidence, and even the risk of gastrointestinal malignancy and pneumonia, with moderate levels of evidence [76].

B. STOPP/START-STOP Criteria—STOPP, the Screening Tool of Older Person’s Potentially Inappropriate Prescriptions, and START, the Screening Tool to Alert Doctors to Right Treatment [77] tool was realized by 18 experts in geriatric pharmacotherapy in Europe and which aims at the list of recommendations that warns on some criteria for initiating or stopping a treatment taking into account the existing comorbidities. These criteria also take into account the possible “inappropriate medication prescribing (IMP) in the older adults (STOPP criteria)” [77] and “potential missed prescribing (PPO)” (START criteria) [8]. The STOPP/START criteria aim to improve the rationale for drug administration, reduce polypharmacy and the number of adverse effects by about 70–80% and reduce costs. Regarding the use of PPIs in the older adults, the recommendations of the latest version (Delphi version 3) support the same recommendations as the Beers criteria (F2,F5,H1) and reject a STOPP criterion, i.e., “Aspirin with a history of peptic ulcer disease without concomitant proton pump inhibitor (risk of recurrent peptic ulcer)” [77].

C. FORTA List (Fit for the Aged), used in Europe in 12 countries, classifies drugs into four classes. The EURO FORTA list of 2018, contains 286 drugs grouped into 30 diseases or syndrome [78], respectively, “class A (A-absolutely), indispensable drug, with proven benefit on the efficacy/safety ratio in older adult patients for a given indication, class B (B- beneficial), drugs with proven efficacy or efficacy in the older adults, but limited in terms of effect or safety, class C (C-can’t), drugs with questionable safety and efficacy profile in the older adults, to be avoided or omitted in older adult patients due to polypharmacy, lack of evidence of benefit and (it is recommended to review/ find alternatives) and class D (D-don’t),drugs to be avoided in the older adults, preferably to be omitted and find alternatives” [78]. In addition, these criteria also contribute to an optimization in terms of treatment, taking into account the “anatomo-therapeutic-clinical classification of drugs” [5,78]. In this case, the reference with respect to PPIs is that there is in practice overtreatment with PPIs in patients with gastro-esophageal reflux disease. In practice, the list can be accessed at the link https://forta.umm.uni-heidelberg.de or via the application on mobile devices—the FORTA application—for Android and IOS, a bilingual application (German and English) that is available free of charge. In these conditions, there is a concern to reduce, as much as possible, the excessive use of PPIs, especially among older adult patients, but not only for them, with the concern about the occurrence of adverse reactions also being for other age groups. There is talk about the gradual introduction of a policy of “reducing inappropriate prescribing”, but also of “deprescribing” PPIs, respecting certain steps that can be followed by both physicians and pharmacists [79,80]. One such tool is the STRIP Guide (The Systematic Tool to Reduce Inappropriate Prescribing), developed in the Netherlands, with the aim of reducing “inappropriate prescribing of medicines to the older adults” and comprising “5 steps”, namely “medication assessment, review of the existing therapeutic plan, consultation with the patient to establish the patient’s medical goals, development of the therapeutic plan with the aim of reaching agreement between the doctor and the patient on the goals of treatment, and follow-up and monitoring” [5,81].

We propose a possible decision process for deprescribing PPI’s in older adults. The foremost idea is the fact that deprescribing should take into consideration the particularities of each patients, as a starting point. In order to achieve this, an individualized plan that the patient can understand and accept is a must. This plan should be carefully revised every six months and should evaluate the risks of the patients for adverse reactions, medicines, and alimentary interactions, together with the facility state. Based on this, an assessment of PPI current indication is necessary, and if no clear indication exists, PPI administration should stop. At the same time, if PPIs are needed, the length of administration should be evaluated, and if this is longer than 8 weeks, PPI administration should stop. Of course, if there are any clear indications, considering geriatric guidelines for PPI administration, these should continue. Another aspect to evaluate is related to symptoms persistence. Ideally, PPI should have a progressive dose reduction, with a careful reevaluation of the risks for rebound hypersecretion.

## 8. Strengths and Limitations

The study offers a comprehensive synthesis of the current evidence on the topic. By synthesizing data from various studies, published in peer-reviewed journals, the study offers a holistic perspective on the clinical use of PPIs among older adults, a vulnerable population. The study evaluates the risks associated with long-term PPIs use, with findings of significant importance for healthcare providers and focus on geriatric patients. The broad analysis captures a wide range of situations that doctors and patients alike meet in real life.

The major limitation is that it does not follow a systematic approach, without conducting an assessment for the risk of bias or any quantitative analysis. Also, the inclusion of only the English language sources may limit global reach and potentially particular situations. Finally, the heterogeneity of the included studies and the specific of the methods used for analysis limit the generalizability of the findings.

Considering the wide use of PPIs in older adult patients with comorbidities, we consider that future research should focus on the continuous monitoring of clinical benefits and potential risks. This would offer the possibility for a fast and correct response if new data is generated. Also, considering the associated risks, studies on the use of technology for support and monitoring for older adult people can improve their quality of life, limit the overuse of PPIs, and decrease the risk for long-term adverse reactions.

## 9. Conclusions

The medium- and long-term use of PPIs is a certainty, proven by various statistics around the world. Elderly patients with comorbidities are the most exposed to inappropriate use of this class of drugs, which, it should be noted, is extremely useful when administered appropriately, following the recommendations of clinical guidelines in force. The inappropriate prescribing of PPIs can contribute to polypharmacy, decreased patient compliance with treatment, the development of drug interactions, adverse effects that are sometimes neglected and produce their effects over time, “cascade prescribing” and, last but not least, an increased risk of decompensation, with increased visits to emergency departments or/and hospitalizations. Therefore, a thorough and comprehensive evaluation of each patient is essential, with a focus on identifying all underlying pathologies and associated risks.

While the current prescribing practices follow clinical guidelines, a significant limitation remains: although guidelines specify when to initiate proton pump inhibitor therapy, they do not always clearly indicate when to discontinue treatment, considering the individual risk–benefit profile of each patient. Addressing this gap must include clear recommendations related to regular reviews of the medication, periodic treatment evaluations using standardized criteria, reducing inappropriate prescription and use, and even deprescribing PPIs when there is no ongoing indication or strong evidence of benefit.

Future considerations should take into account pharmacogenetics and personalized medicine, with a focus on the PPIs metabolism and therapeutic response, which may vary based on the patient-specific genetic profile. Therapeutic alternatives that are recently introduced on the market, such as potassium-competitive acid blockers, could be a viable alternative when PPIs have limitations.

Educational interventions and strategies aimed at improving prescribing practices, medication adherence, and the awareness of deprescribing may optimize patient safety and treatment efficacy. The general practitioner, in collaboration with the geriatrician, plays a key role within the multidisciplinary team by synthesizing the diagnosis and treatment plan for the older adult patient. Collaboration between physicians of different specialties for the benefit of patients is very important, with the common goal being to ensure appropriate, personalized, patient-centered treatment and an increase in the quality of life.

## Figures and Tables

**Table 1:** Summary of drug interactions involving proton pump inhibitors. (↓—decreases; ↑—increases).

Drug/Class	Interaction with PPI	Mechanism	Clinical Implication
Tyrosine kinase inhibitors (Danetimib, Erlotinib)	↓ Absorption	Increased gastric pH	Reduced efficacy
Protease inhibitors (Atazanavir, Indinavir)	↓ Absorption	Increased gastric pH	Reduced antiviral activity
Antifungals (Ketoconazole, Itraconazole)	↓ Absorption	Increased gastric pH	Reduced antifungal efficacy
Oral iron preparations	↓ Absorption	Reduced solubility due to increased pH	Iron deficiency risk
Clopidogrel	↓ Activation	CYP2C19 inhibition	Reduced antiplatelet efficacy
Prasugrel, Ticagrelor	No interaction	Not dependent on CYP2C19	Safe co-administration
Methotrexate	↓ Elimination	Delayed clearance	Increased toxicity risk
Phenytoin	↑ Levels	CYP2C19 inhibition	Toxicity (CNS symptoms)
Diazepam	↑ Levels	CYP2C19 inhibition	Prolonged sedation
Carbamazepine	↑ Levels	CYP3A4 inhibition	Risk of toxicity
Cyclosporine	↑ Levels	CYP3A4 inhibition	Nephrotoxicity risk
Vonoprazan (P-CAB)	N/A	Not affected by CYP2C19	Alternative in PPI resistance
